# Supplementary material for: Chromothripsis during telomere crisis is independent of NHEJ, and consistent with a replicative origin
Source: Genome Res. 2019 May;29(5):737–49. doi: 10.1101/gr.240705.118 (PMC6499312; doi:10.1101/gr.240705.118)
Supplement: Supplemental Material [file supp_gr.240705.118_Supplemental_file_1.zip › contigs/annotated_contigs/DB54/contig.2.DB54_length_380_mean_cov_13.9105263158.docx]

**DB54_length_380_mean_cov_13.9105263158**

```
GGCGGGAAGATAAGGAGGAAAAAAAAACAAAACAAACCTGG|AAAAAAAAATAAAACCCAACATAAAAAAAAAAAA|AAAAAAAA|AAA
                                          >chr9:27922106-27922148 + E=9e-05 p=0e+00 >c
AAAAAAAAATTAGCCAGGTGTGGTGGCGGGCACCTGTAGTTCCAGCTACTAGGGAGGCTGAGGCAGGAGAATGGCGTGAACCTGGGAGG
hr1:193314567-193314847 + E=2e-152
CGGAGCTTGCAGTGAGCCGAGATGGCGCCACTGCACTCCAGCCTGGGCGACAGAGCGAGACTCCGTCTCAAAAAAAAAAAAAAATCCTG

TGCTAAAAAGATGTGTCACATTACAGTTTTATAGGAGCAGCTTTAGTACTGGGAATTGGAATAGGAGCTGTTTCAATACATTCTAATTT

A|TTTTTGGTTTTGGGGGGGAAATTGTG
```
